# Clinical Management of Cervical Restorations with Closing Gap Technique: A Follow-Up of Two Cases

**DOI:** 10.3390/dj14010013

**Published:** 2026-01-01

**Authors:** Alexander Bonchev

**Affiliations:** Department of Conservative Dentistry, Faculty of Dental Medicine, Medical University, Sofia, 1431, Bulgaria; a.bonchev@fdm.mu-sofia.bg

**Keywords:** cervical lesions, resin composite, NCCLs, marginal adaptation, Closing Gap Technique

## Abstract

**Background:** Cervical restorations remain clinically challenging due to complex anatomy, limited enamel availability, and difficulties in achieving reliable adhesion at dentin or cementum margins. Polymerization shrinkage and marginal leakage are frequent causes of failure. Although the Closing Gap Technique has been proposed to improve marginal adaptation in cervical restorations, evidence supporting its medium- to long-term clinical performance is limited. The aim of this case report was to evaluate the clinical effectiveness of the Closing Gap Technique in the restoration of carious and non-carious cervical lesions. **Materials and Methods:** Two patients presenting with cervical lesions were treated using the Closing Gap Technique. One case involved carious cervical lesions, while the second included multiple non-carious cervical lesions. Restorations were performed following an enamel-anchored incremental layering protocol with resin composite. Clinical evaluations were conducted at 8 years (case #1) and 2 years (case #2) post-treatment, respectively. **Results:** Both cases demonstrated favorable clinical outcomes at follow-up. The restorations exhibited good marginal integrity, satisfactory esthetics, absence of marginal discoloration, no secondary caries, and no signs of debonding. The only minor defect observed was slight chipping of one of the restorations. **Conclusions:** Within the limitations of this case report, the Closing Gap Technique showed stable and predictable medium- and long-term clinical performance, supporting its use as a viable restorative approach for managing cervical lesions in daily clinical practice.

## 1. Introduction

Cervical cavities represent a significant clinical challenge due to their complex morphology and anatomical location. These lesions generally have a non-retentive configuration, with margins that frequently extend onto dentin or cementum. There areas provide weaker adhesion compared to enamel and are situated near the gingival margin, making the moisture control difficult [1,2]. Both carious and non-carious cervical lesions (NCCLs) can appear in this region, each having unique causes and structural characteristics that affect the restorative strategy. Such defects are frequently linked to gingival recession and poor oral hygiene practices [3]. The occurrence of cervical caries increases with age, especially in individuals over 50 years old, due to long-term exposure to factors such as periodontal disease and exposed cervical dentin [3]. Management of cervical lesions may involve conservative restorative procedures or surgical treatments, such as subepithelial connective tissue grafting, depending on the extent and severity of the lesion [3,4,5,6].

One of the main challenges in treating such lesions is establishing a durable marginal seal. Poor sealing can result in microleakage, which in turn may cause discoloration at the margins, secondary caries, and reduced restoration lifespan [1,7]. The effectiveness and longevity of resin composite restorations are therefore closely dependent on achieving strong adhesion to tooth structures [1,8,9,10].

Several restorative materials have been recommended for treating NCCLs, including glass ionomer cements, resin-modified glass ionomers, compomers, and resin composites of different consistencies [11,12]. Among these, resin composites are often preferred due to their excellent esthetics, mechanical durability, and resistance to wear [13,14,15]. In addition to material selection, factors such as cavity design [16], the adhesive system used, and the layering technique applied all significantly influence the long-term success and stability of cervical composite restorations [17,18].

Furthermore, the method of application represents another key element influencing the prognosis of cervical restorations. Because of the anatomical complexity of the cervical area, these restorations often require specific clinical approaches, and the success of these techniques can differ between practitioners. Studies indicate [4,19] that implementing modern, innovative techniques helps to reduce common procedural errors and enhances the overall clinical outcome. Among the various restorative strategies proposed for managing cervical lesions, the “Closing Gap Technique”, introduced in 2012, represents an alternative approach [20]. This layering protocol was specifically designed to minimize polymerization shrinkage-related stress by utilizing the strong bond between composite resin and enamel as a foundational base for building the restoration in successive layers toward the cervical margin. Through this approach, the technique aims to enhance marginal adaptation and long-term stability of the restoration in areas that are otherwise challenging to seal effectively.

Although the Closing Gap Technique is not new, the available literature provides limited evidence regarding its medium- to long-term clinical performance. To date, only one clinical report has documented a follow-up of two years involving three cervical restorations performed using this technique [20].

The aim of the study is to explore the effectiveness of the Closing Gap Technique in the restoration of both carious and non-carious cervical lesions through two clinical cases. Follow-up evaluations were conducted at 8 years post-treatment for the first presented case, representing a long-term outcome not previously documented in the literature, and at 2 years post-treatment for the second case. By providing additional medium- and long-term clinical evidence on the performance of this technique, the present report aims to contribute to the existing literature and support informed clinical decision-making in the management of cervical lesions.

## 2. Materials and Methods

### Description of the Closing Gap Technique

**Figure 1:**
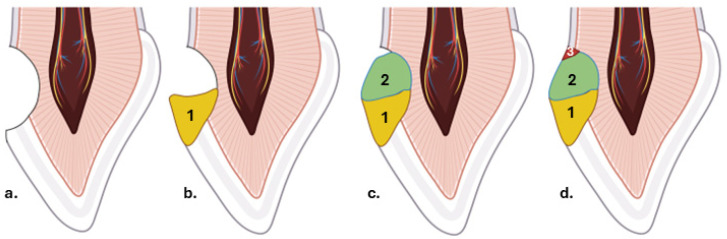
Step-by-step illustration of the closing gap incremental technique: (**a**) Prepared cervical cavity with beveled enamel margins to enhance adhesion of the initial composite layer; (**b**) placement of the first layer over the enamel (1), extending across the bevel and following the natural vestibular contour; (**c**) application of the second layer (2), intentionally leaving a small cervical gap of approximately 1 mm; (**d**) final “closing of the gap” achieved by placing and adapting the last composite increment (3).

To minimize the effects of polymerization shrinkage, the “Closing Gap Technique” employs incremental layering of the composite resin in progressively smaller portions, starting from the coronal margin and proceeding toward the cervical area, intentionally leaving a narrow space of approximately 1 mm. Each increment is applied to adhere to two surfaces, one being the dental substrate and the other the underlying composite layer. While there is no fixed number of layers required, the cavity’s size and shape, as well as the consistency of the composite material, determine the number of increments necessary for complete restoration. The final step involves filling the remaining gap, thereby completing the restoration [20] (Figure 1).

**Figure 2:**
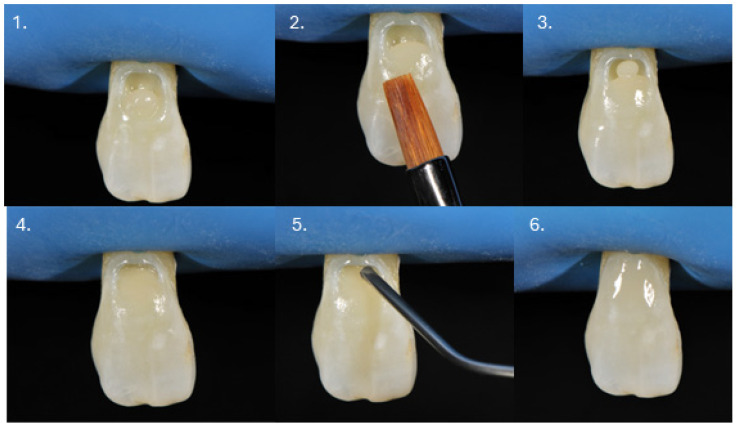
Procedure sequence of the restorative technique. (1) Application of the first resin composite layer into the cavity; (2) use of a composite brush to enhance adaptation and contouring of the composite material; (3) application of the second composite layer; (4) careful adaptation of the second layer to the cavity walls; (5) application of a flowable composite (optional) as the final layer; and (6) completed restoration.

The described technique allows the final layering to be performed using either a conventional (regular) or flowable composite (Figure 2). In the cases presented, and according to the author’s preference, the restorations were completed entirely with a conventional composite material.

The assessment of the reported cases was primarily qualitative and based on clinical examination and photographic documentation. Standardized clinical evaluation criteria were not applied due to the descriptive nature of this case report, the limited number of cases, and the retrospective design of the long-term follow-up, which did not allow for systematic scoring at baseline. Nevertheless, careful clinical observation focusing on marginal integrity, esthetics, retention, and absence of secondary caries was performed to evaluate the restorations’ performance over time.

## 3. Results

### 3.1. Presentation of Clinical Case #1

#### 3.1.1. Patient Information

A 34-year-old female patient with no systemic diseases or relevant medical history was referred to the dental office in 2017 for the replacement of cervical composite restorations on teeth 34 and 44 due to unsatisfactory esthetics. The patient reported no pain or sensitivity in the affected area, and no history of parafunctional habits was declared.

#### 3.1.2. Clinical Findings and Diagnosis

**Figure 3:**
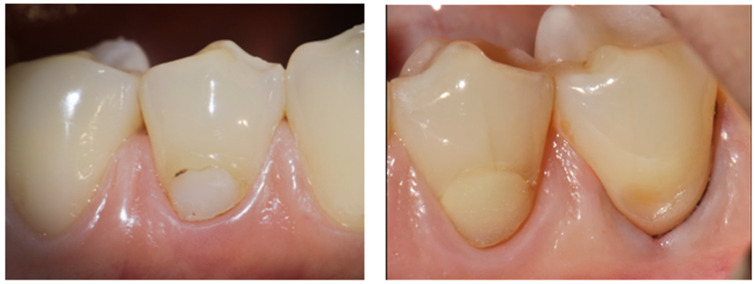
Intraoral view showing existing cervical composite restorations on teeth #34 and #44 with marginal discoloration, microleakage, and chipping; a U-shaped non-carious cervical lesion on tooth #35.

Intraoral examination revealed existing cervical composite restorations on teeth 34 and 44, exhibiting marginal discoloration, visible microleakage, and chipping along the gingival margins. The composite material demonstrated notable loss of surface luster and poor adaptation to the cavity walls, suggesting material deterioration over time. The surrounding gingival tissues appeared healthy, with no signs of inflammation or bleeding on probing and occlusal wear facets were observed. Tooth 35 presented a non-carious cervical lesion with a characteristic U-shaped morphology. Tooth 45 had been previously restored with a composite restoration using the Closing Gap technique, showing satisfactory adaptation and surface integrity (Figure 3).

#### 3.1.3. Therapeutic Intervention

Before restorative treatment the patient received professional oral prophylaxis consisting of supragingival scaling using ultrasonic instruments (Woodpecker, Guilin, Guangxi Province, China), followed by polishing with a rubber cup (Pro-Cup™ prophylaxis cups, Kerr Corporation, Orange, CA, USA) and fluoride-free prophylactic paste (Proxyt fluoride-free, Ivoclar, Schaan, Liechtenstein). Oral hygiene instructions were given, and the restorative procedures were performed after ensuring satisfactory plaque control and gingival health.

**Figure 4:**
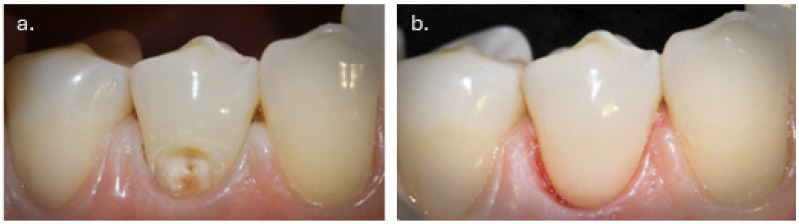
(**a**) Cavity on tooth 44 after removal of the previous composite restoration and caries excavation, with the enamel margin beveled. (**b**) Final result of the restoration completed using the Closing Gap technique.

After removal of the existing composite restorations on teeth 34 and 44, secondary caries lesions were identified and thoroughly excavated. A short enamel bevel (45°, 1–2 mm) was prepared on the enamel margins using a diamond round taper bur (fine-grit, Axis, Crissier, Switzerland). The teeth were isolated with a rubber dam prior to the adhesive procedure. Isolation was performed gently around the restored teeth using only ligatures and an inverted medium rubber dam sheet (Nic Tone, Zapopan, Jalisco, Mexico), without the use of metal clamps. A total-etch protocol was perfomed using 37.5% phosphoric acid gel (Kerr Corporation, Orange, CA, USA), with 15 s of etching for dentin and 30 s for enamel, followed by rinsing the etchant for over 30 s. The adhesive system (OptiBond FL, Kerr Corporation, Orange, CA, USA) was applied according to the manufacturer’s instructions. The primer was applied using a microbrush (POINT applicators, medium size, SDI Limited, Bayswater, Victoria, Australia) and scrubbed into dentin for 15 s with gentle brushing motion, followed by air-drying for 3 s. The adhesive was then applied with the same microbrush using a light brushing motion for an additional 15 s, air-thinned for 3 s, and light-cured for 20 s. The cavities were then restored using a composite resin (Estelite Asteria, Tokuyama Dental Corporation, Tokyo, Japan) employing the Closing Gap technique. The material was applied in two consecutive 2 mm increments (Figure 4). The smaller lesion on tooth 35 was restored using a single increment of composite material. Restorations were performed with LM-arte Applica and LM-arte Condensa instruments (LM-Dental, Parainen, Finland). Polymerization of each increment was carried out using a light-curing lamp with an intensity of 1100 mW/cm^2^ (BluePhase Style, Ivoclar, Schaan, Liechtenstein). The light was positioned 2 mm from the composite surface, and each increment was cured for 10 s, according to the resin manufacturer’s instructions. The restorations were polished under water cooling using polishing rubber system (Diacomp Plus, EVE Ernst Vetter GmbH, Keltern, Germany).

#### 3.1.4. Follow Up and Outcomes

**Figure 5:**
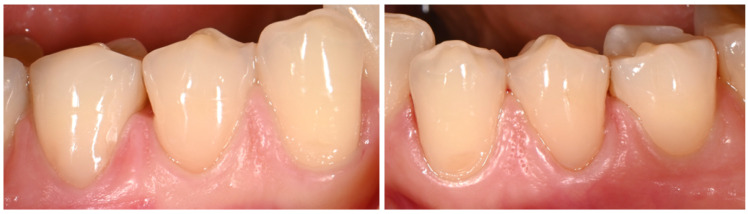
Eight-year follow-up after professional cleaning, showing well-preserved cervical restorations with minor chipping on tooth #45.

Figure 5 presents the eight-year follow-up of the cervical restorations. During this period, the patient resided abroad but attended regular check-ups; however, the composite restorations were neither polished nor replaced. Clinical evaluation of teeth #34, #35, #44, and #45 revealed excellent restoration integrity, with preserved surface luster, no marginal discoloration, and absence of secondary caries. The restorations maintained proper function, with no detectable wear, fractures, or hypersensitivity reported by the patient. The only minor defect observed was slight chipping of the restoration on tooth #45, which could be easily repaired. A reduction in the height of the interdental papillae between teeth #44–#45 and #34–#35 was noted, likely due to suboptimal contouring of the approximal restorations. Therefore, a replacement of these restorations was advised. Cervical erosion was observed on tooth #33, and a composite restoration was recommended for this tooth.

### 3.2. Presentation of Clinical Case #2

#### 3.2.1. Patient Information

A 40-year-old male patient presented to the dental office with a chief complaint of unsatisfactory dental esthetics, primarily due to generalized erosive lesions. His main concern was the pronounced yellowish discoloration associated with these defects. The patient reported regular weightlifting training, daily consumption of water mixed with lemon juice over several months, and vigorous horizontal tooth-brushing. He also disclosed a history of smoking. No symptoms of hypersensitivity were reported in the teeth affected by the NCCLs.

#### 3.2.2. Clinical Findings and Diagnosis

During clinical examination, NCCLs were identified on teeth #16–#12, #22–#25, #35–#34, and #43–#45. The largest lesions displayed features consistent with mechanical abrasion, including distinct grooves and localized gingival recession, while signs of biocorrosion (erosion) were evident, characterized by sharply demarcated enamel margins. Most lesions exhibited a U-shaped morphology. The exposed dentin surfaces were covered with brown pigmentation, which was easily removed by polishing and was likely associated with the patient’s smoking habit. Occlusal wear facets with erosive cupping were also present. The patient’s diet, which included abrasive foods such as nuts, may have further contributed to tooth surface loss. Collectively, these clinical findings indicate a multifactoral etiology of the observed NCCLS.

**Figure 6:**
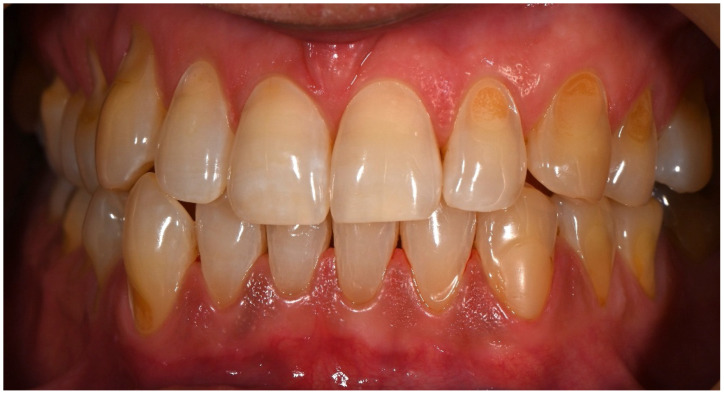
Intraoral photograph showing multiple non-carious cervical lesions with characteristic U-shaped morphology, brown dentin pigmentation, and sharply demarcated enamel margins, consistent with combined abrasion–erosion etiology.

The yellowish appearance of the lesions which was the patient’s primary esthetic concern, was attributed to the presence of sclerotic dentin. Additional erosive defects were observed on teeth #11, #21, and #32. However, these lesions were less than 1 mm in depth and confined to enamel without dentin involvement. Vitality testing showed positive responses for all affected teeth. Based on these findings, a diagnosis of NCCLs requiring restorative intervention was established (Figure 6).

#### 3.2.3. Therapeutic Intervention

At the beginning of treatment, the patient was advised to reduce or discontinue the intake of highly acidic foods and beverages, including diluted fresh lemon juice, and was instructed in proper tooth-brushing technique to minimize further abrasion. After cleaning and polishing the teeth using an air-flow device, the patient elected to undergo a home bleaching protocol. A 10% carbamide peroxide gel (Opalescence, Ultradent Products, Inc., South Jordan, UT, USA) was applied with overnight tray wear for a period of two weeks. Three weeks after completion of the whitening procedure, the final tooth shade was stabilized, and restorative treatment of the NCCLs was initiated.

Following isolation and placement of a retraction cord, the dentin surfaces of the lesions were roughened with a coarse round diamond bur (Strauss & Co., Raanana, Center District, Israel), and the enamel margins were beveled. A total-etch protocol was performed, as desribed in Section 3.1.3. consisting of 15 s of etching for dentin and 30 s for enamel (37.5% phosphoric acid gel Kerr Corporation, Orange, CA, USA). The etchant was thoroughly rinsed, and a two-bottle adhesive system (Kerr Corporation, Orange, CA, USA) was applied according to the manufacturer’s instructions.

**Figure 7:**
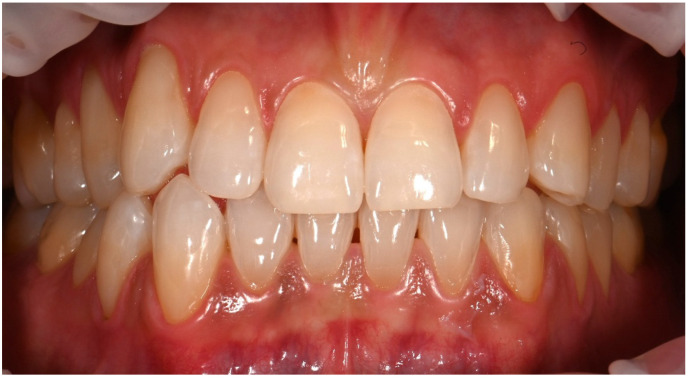
One-week postoperative clinical view showing the final esthetic outcome after restoration of the non-carious cervical lesions, demonstrating proper integration of the composite restorations with the surrounding tooth structure and stable gingival conditions.

Most lesions were restored with a universal composite resin (Tetric Prime, Ivoclar, Schaan, Liechtenstein) using the previously described “Closing Gap Technique,” except for tooth #12, where the smaller lesion size did not require this approach. Restorations were performed with OptraSculpt instruments (Ivoclar, Schaan, Liechtenstein). Polymerization of each increment was carried out using a light-curing lamp with an intensity of 1100 mW/cm^2^ (BluePhase Style, Ivoclar, Schaan, Liechtenstein). The light was positioned 2 mm from the composite surface, and each increment was cured for 10 s, according to the resin manufacturer’s instructions. The existing composite restoration on tooth #33 was recontoured and polished to harmonize with the adjacent restored surfaces. After placement, all restorations were polished using OptraGloss polishing rubbers (Ivoclar, Schaan, Liechtenstein) under water cooling to achieve a high-gloss finish and optimal surface integration (Figure 7).

#### 3.2.4. Follow Up and Outcomes

**Figure 8:**
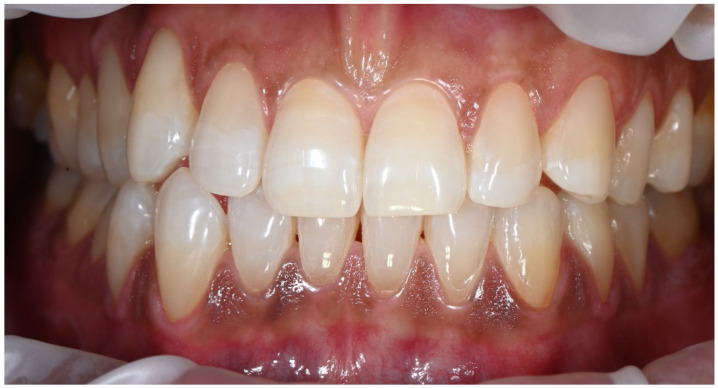
Two-year follow-up showing stable integration of the cervical restorations, with maintained anatomical form and healthy gingival tissues despite slight surface luster reduction.

The intraoral photograph presented in Figure 8 illustrates the two-year follow-up of the restorative procedures, taken during a routine check-up appointment. Throughout the follow-up period, the patient reported consistent use of a soft-bristle toothbrush and adherence to proper brushing technique. He also avoided highly acidic foods and beverages and reduced coffee consumption. Smoking, however, remained a persistent part of his lifestyle, which may continue to influence long-term restorative and periodontal outcomes. Over this period, the restorations had not undergone additional polishing. A slight reduction in surface luster of the composite material was observed; however, the overall integration of the restorations remained satisfactory, with no evidence of marginal discoloration, microleakage, chipping, secondary caries, or material degradation. The anatomical form and contour were well maintained, and the gingival tissues exhibited healthy appearance with no signs of inflammation or worsening the recession.

## 4. Discussion

The cervical area presents a set of anatomical, biomechanical, and adhesive challenges that uniquely influence restorative outcomes, making it a critical focus in restorative dentistry. Because the cervical margin often lies in regions with little to no enamel or directly on root dentin, bonding procedures become more complex, increasing the risk of marginal leakage and ultimately compromising restoration longevity. In vitro evidence indicates that the presence of cervical enamel significantly improves the behavior of composite restorations by enhancing fracture resistance, thereby contributing to more predictable clinical performance. For these reasons, restorative strategies in the cervical region must be adapted to its distinct structural and functional characteristics. Careful selection of restorative materials with appropriate mechanical properties is essential to ensure favorable biomechanical behavior and long-term durability of restorations [21].

Based on the clinical findings and the patient’s anamnesis in clinical case number two, the non-carious cervical lesions (NCCLs) in this case appear to result from a combination of abrasion, primarily due to improper horizontal tooth-brushing with excessive force, and erosion associated with frequent consumption of lemon juice diluted in water. The lesions vary in size and exhibit a characteristic U-shaped morphology. The current literature provides limited evidence that NCCLs arise from abrasion alone, indicating that these lesions are typically multifactorial in origin [22]. The patient’s chief complaint in the present case was the compromised esthetic appearance of the lesions, particularly their yellowish discoloration. Owing to the chronic and slowly progressive nature of the NCCLs, the patient did not report any symptoms of hypersensitivity. Although the progression rate of NCCLs is relatively slow [23], it can vary considerably among individuals. Consequently, it is essential to identify and reduce the etiological factors while establishing an individualized monitoring protocol [24].

In this case, the patient demonstrated good compliance by modifying tooth-brushing habits, adopting a soft-bristle toothbrush, and discontinuing the consumption of lemon water. As a result, the lesions on teeth #11, 21, 31 were managed conservatively and placed under periodic observation rather than restored immediately. The current literature suggests that NCCLs with a depth of less than 1 mm are typically monitored at regular intervals rather than treated with restorative materials [25,26]. Standardized intraoral photographs and dimensional measurements of the lesions were obtained for follow-up. According to the findings of Du et al., NCCLs with a depth of approximately 1.5 mm should be restored using an adhesive resin system to prevent further structural deterioration and to maintain normal tooth function [27]

Non-carious cervical dentin defects exhibit distinct structural characteristics compared to sound dentin, primarily as a consequence of prolonged exposure to the oral environment [17,28]. Continuous contact with saliva promotes the development of a hypermineralized surface layer [29]. This altered dentin is marked by elevated phosphate content, reduced carbonate levels, an increased degree of crystallinity [30]. As a result of these sclerotic changes, the adhesion of composite resins to the affected dentin substrate is often compromised, potentially contributing to a greater likelihood of restoration loss over time [17,31].

Dentin found in cervical lesions often exhibits a dense, hypermineralized surface with blocked dentinal tubules [17,32]. These altered structures are resistant to complete removal through etching, leading to poor dentin hybridization if they remain [33]. In the reported cases, low-speed cutting with a diamond bur was used to mechanically roughen the non-carious cervical lesions. This approach was chosen because total-etch adhesive systems, which eliminate the smear layer, perform better on the rougher surface produced by a diamond bur, whereas carbide burs are typically recommended for self-etch systems that modify rather than remove the smear layer [34]. Clinical studies have indicated that additional surface roughening can sometimes reduce restoration failure rates [32]. This improvement is attributed to the mechanical removal of sclerotic dentin, allowing for the development of a more effective hybrid layer [35]. The American College of Prosthodontists also advises creating a textured surface on sclerotic dentin using a rotary instrument to enhance restoration retention. It further recommends selective enamel beveling to increase the available bonding surface, as it was performed in the presented cases. Beveling the enamel in cervical restorations serves to increase the available bonding surface, promote micromechanical retention, and improve the esthetic integration of the restoration by facilitating a more gradual transition in color between the restorative material and the natural tooth structure [36]. Enamel is more effectively etched than dentin, and beveling exposes more enamel prisms at an optimal angle, which facilitates better adhesive penetration and bond strength [37]. However, evidence from systematic reviews and randomized controlled trials shows that beveling can enhance early retention and marginal adaptation, but its long-term advantages over non-beveled margins remain unclear [38,39,40]. Either multistep (etch-and-rinse) or single-step (self-etch) adhesive systems may be used. For sclerotic dentin in particular, the etching time can be extended to approximately 30 s, instead of the traditional 15 s and the adhesive should be actively scrubbed for about 20 s [36].

Secondary caries are a major reason for restoration failure and occur most often at the gingival margins of class II and class V restorations [41]. Resin composites, glass ionomer cements (GICs), resin-modified glass ionomers (RMGICs), and compomers are commonly used for cervical caries, and the American Dental Association recommends all of them as suitable direct restorative materials, with the choice depending on factors such as moisture control, esthetic needs, and individual patient conditions [42,43].

A recent systematic review by Chaple Gil et al. [4], which included seventeen randomized controlled trials, reported that conventional composites used with two-step etch-and-rinse adhesives and compomers showed the highest retention in pairwise comparisons. GICs demonstrated the most consistent long-term results. However, the network meta-analysis found no statistically significant differences among the materials. Although compomers ranked highest overall, the supporting evidence showed considerable heterogeneity and a high risk of bias [4].

The Closing Gap Technique offers several clinical advantages, including guided buccal contouring, the possibility of early shade verification, minimal polymerization stress, and enhanced control over composite placement [20]. Nevertheless, a study by Correia et al. [18] demonstrated that using an incremental layering technique with oblique increments, beginning from the gingival margin, resulted in lower polymerization shrinkage stresses in Class V cavities of maxillary premolars, as shown through three-dimensional finite element analysis. Interestingly, the same study reported that the best stress distribution pattern was achieved in the model restored using a bulk-fill resin technique. This finding is further supported by the study conducted by Correia et al. [18], which demonstrated that bulk-fill composites may produce lower polymerization shrinkage stresses at both the gingival and incisal margins when compared with incremental layering techniques. Nevertheless, despite the continuous improvements in the aesthetic properties of bulk-fill composites, their optical characteristics still do not fully match those of conventional composite resins [44]. A recent study by Dhammayannarangsi et al. [21] revealed that bulk-fill flowable composites can reduce interfacial stress and may improve adaptation to cavity walls, their long-term wear resistance and color stability still need further clinical validation. Furthermore, clinical observations from cases performed approximately eight years ago revealed that earlier generations of bulk-fill composites exhibited noticeably inferior aesthetic outcomes compared to modern formulations. This was the main reason why the presented clinical cases were restored using an incremental layering technique and a universal composite resin. However, this choice reflects the clinician’s preference based on aesthetic considerations at the time of treatment rather than a definitive clinical superiority of the incremental layering technique over bulk-fill approaches.

The guided buccal contouring achieved with this composite placement technique supports the creation of an appropriate emergence profile of the tooth. This is the critical interface between the restoration and the gingiva, which contributes to stable gingival architecture and improved esthetic outcomes [45]. It supports effective plaque control by allowing the lips, cheeks, and tongue to naturally cleanse the restoration surfaces, helps maintain gingival health by avoiding overcontouring that can trap plaque and cause inflammation, and ensures that the restoration reproduces the tooth’s natural anatomy for optimal esthetic and functional results [46].

Moreover, cervical cavities bordered by enamel at the incisal margin and dentin at the cervical margin are subjected to flexural forces due to the substantial difference in elastic moduli between these two dental tissues (enamel: approximately 84.1 GPa; dentin: 16.6–18.6 GPa) [17]. Consequently, the restorative material selected must possess mechanical properties capable of accommodating this disparity [17,47]. Some authors have suggested that restorative materials with a lower elastic modulus can better accommodate tooth flexure, potentially enhancing the retention of Class V restorations compared to materials with higher rigidity. However, recent clinical studies evaluating Class V restorations made with composites of varying elastic moduli have reported no significant differences in retention rates between the materials [2,48]. Additionally, teeth exhibiting occlusal wear facets were found to have a higher risk of restoration failure compared to teeth without such features. The presence of occlusal wear facets is typically linked to heavy occlusal loading or parafunctional habits, which generate concentrated stress in the cervical region. Repeated cycles of compressive and tensile forces from tooth flexure can therefore contribute to marginal breakdown and loss of cervical restorations [49,50].

Rubber dam isolation provides a dry operative field, which is critical for the success of adhesive and restorative procedures, as moisture contamination can compromise bond strength and the integrity of restorations [51]. However, the use of a rubber dam with dental clamps during cervical restoration placement may contribute to the occurrence or progression of gingival recession [52]. Moreover, it has been recommended by Faveti et al. [52] that isolation of the target tooth with cotton rolls should be used whenever possible. In the presented cases, a rubber dam was employed without a clamp, secured with ligatures when necessary, and applied with very gentle handling to minimize potential trauma.

### 4.1. Limitations of the Study and the Closing Gap Technique

The assessment of the reported cases was primarily qualitative, based on clinical examination and photographic documentation. Standardized evaluation criteria could not be applied due to the descriptive nature of this case report, the limited number of cases, and the retrospective design of the long-term follow-up, which precluded systematic baseline scoring.

It should be noted that the patient good compliance with behavioral modifications, including adopting a soft-bristle toothbrush, adjusting brushing technique, and discontinuing the consumption of lemon water may have independently contributed to the positive clinical outcome and act as potential confounding factors in evaluating the effect of the restorative technique alone.

One limitation of the closing gap technique is the increased risk of contamination from gingival fluid, as the gingival portion of the cavity is restored last. Such contamination during adhesive procedures can compromise marginal adaptation and elevate microleakage, especially if it occurs after the primer or adhesive has been applied. This, in turn, may raise the likelihood of restoration failure and the development of secondary caries [53].

The presented cases provide additional clinical information on the Closing Gap Technique and reflect the authors’ interpretation of its application. However, they do not allow for definitive conclusions regarding the suitability of this technique for all clinical scenarios, as no statistical analysis was performed.

### 4.2. Clinical Significance of the Study and Future Perspectives

This follow-up cases provides long-term (8-year) clinical data on cervical restorations performed using the Closing Gap Technique, which, to our knowledge, have not been previously reported in the literature. Moreover, a follow-up reporting outcomes for as many as 14 non-carious lesions restored with this technique has not been previously published. Providing additional clinical information on the performance of this approach may inform clinical decision-making and offer an alternative strategy for clinicians in daily practice.

Further studies involving a larger patient cohort with standardized inclusion and exclusion criteria are warranted. Such research would allow for statistical comparison of different restorative techniques, with the ultimate goal of establishing evidence-based guidelines for the optimal composite placement technique in the cervical cavities.

## 5. Conclusions

The Closing Gap technique demonstrated stable and favorable clinical outcomes in both carious and non-carious cervical lesions, with consistent performance documented at 8-year and 2-year follow-up evaluations. These results underscore the technique’s potential to provide predictable and long-lasting restorative outcomes across different clinical scenarios.

## Data Availability

The original contributions presented in this study are included in the article. Further inquiries can be directed to the corresponding author.

## Abbreviations

The following abbreviations are used in this manuscript:

NCCLs	Non-caries cervical lesions
